# Innate Immune Detection of Cardioviruses and Viral Disruption of Interferon Signaling

**DOI:** 10.3389/fmicb.2018.02448

**Published:** 2018-10-12

**Authors:** Eric C. Freundt, Melissa Drappier, Thomas Michiels

**Affiliations:** ^1^Department of Biology, The University of Tampa, Tampa, FL, United States; ^2^de Duve Institute, Université catholique de Louvain, Brussels, Belgium

**Keywords:** cardiovirus, picornavirus, interferon, dsRNA, innate, RNase L, PKR, MDA5

## Abstract

Cardioviruses are members of the *Picornaviridae* family and infect a variety of mammals, from mice to humans. Replication of cardioviruses produces double stranded RNA that is detected by helicases in the RIG-I-like receptor family and leads to a signaling cascade to produce type I interferon. Like other viruses within *Picornaviridae*, however, cardioviruses have evolved several mechanisms to inhibit interferon production. In this review, we summarize recent findings that have uncovered several proteins enabling efficient detection of cardiovirus dsRNA and discuss which cell types may be most important for interferon production *in vivo*. Additionally, we describe how cardiovirus proteins L, 3C and L^∗^ disrupt interferon production and antagonize the antiviral activity of interferon effector molecules.

## Introduction

*Picornaviridae* is an important family of single-stranded, positive-polarity RNA viruses that includes >30 genera with over 75 species (Zell et al., 2017). Within *Picornaviridae*, the genus *Cardiovirus* includes encephalomyocarditis virus (EMCV), Theiler’s murine encephalomyelitis virus (TMEV) and Saffold viruses (SAFV). Although EMCV has been described as a potential zoonotic agent, SAFVs are the only cardioviruses known to regularly infect humans, with the vast majority of people showing evidence of infection (Zoll et al., 2009; Carocci and Bakkali-Kassimi, 2012). EMCV has been found to infect over 30 host species and contains one serotype, Mengo virus, which was isolated in 1948 in the Mengo district of Uganda (Dick et al., 1948). TMEV was discovered in 1937 by Max Theiler and is found in wild mice and rats worldwide. TMEV can cause different diseases, depending on the virus strain and host genetics, ranging from fatal encephalitis to a chronic demyelinating disease that has served as a model for multiple sclerosis (Brahic et al., 2005).

**FIGURE 1:**
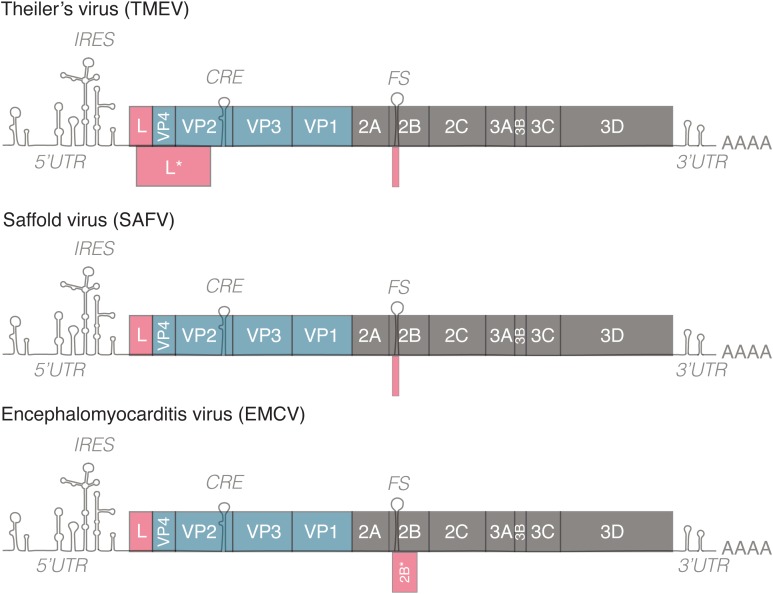
Genomes of representative members of the genus *Cardiovirus*. All members of the genus *Cardiovirus* encode a related leader (L) protein formed by the N-terminal part of the polyprotein. Theiler’s murine encephalomyelitis virus (TMEV) expresses an additional 18 kD protein called L^∗^ from an alternative ORF. TMEV and Saffold virus (SAFV) form the Theilovirus species. These viruses possess a frameshifting site in 2B but the 2B^∗^ ORF is only 14–15 codons in length. EMCV encodes a 128–129 residue-long 2B^∗^ protein. IRES, internal ribosome entry site; CRE, *cis*-replication element; FS, frameshifting site. 5′/3′UTR, 5′/3′untranslated region.

The genome of cardioviruses is approximately 7.8–8.5 kb and contains 5′ and 3′ untranslated regions (**Figure 1**). Translation of the genome gives rise to a polyprotein that is cleaved by the 3C protease, leading to the production of 12 proteins. Two additional proteins, L^∗^ and 2B^∗^, are expressed from alternate open reading frames. L^∗^ is only expressed by TMEV and is important for infection of macrophages, persistence of the virus in mice and inhibiting RNase L (van Eyll and Michiels, 2000; Sorgeloos et al., 2013), 2B^∗^ results from a frameshifting mechanism conserved in cardioviruses that regulates the ratio of structural and non-structural proteins translated over time. Protein 2B^∗^ itself is only thought to be important for replication of EMCV, as mutants that abolish its expression had a small plaque phenotype. 2B^∗^ in TMEV and SAFV is unlikely to act as a protein as it is predicted to encode a peptide of 14 amino acids (Loughran et al., 2011).

In this review, we focus on the ways in which cardioviruses trigger the innate immune response and the efficient mechanisms that they have evolved to suppress these signaling pathways. We consider which cell types may be most important for production of interferon (IFN) *in vivo*, and also describe how cardioviruses disrupt the functions of interferon effectors.

## Induction Of Interferon

Double-stranded RNA (dsRNA) is a necessary product of picornavirus replication, as positive-stranded genome is copied to produce a negative-stranded, full-length template, which is in turn used to produce additional genomes. dsRNA is recognized by several sensor proteins within the cell and triggers a signal transduction pathway that results in transcription of the type I IFN (IFN-α/β) genes, as well as IFN-λ. In the endosome, dsRNA is detected by Toll-like receptor 3 (TLR3), which signals through the adaptor protein TRIF to activate IFN regulatory factor 3 (IRF-3) and nuclear factor kappa B (NF-κB) (Yamamoto et al., 2003). Cytoplasmic dsRNA is detected by the RIG-I-like receptor (RLR) family of proteins, which includes RIG-I (retinoic acid-induced gene I) and MDA5 (melanoma differentiation-associated gene 5). Upon recognition of dsRNA, these proteins undergo a conformational change that exposes N-terminal caspase activation and recruitment domains (CARDs). The RLRs are then capable of stimulating the mitochondrial antiviral signaling (MAVS) protein, also known as IPS-1, Cardif, and VISA, which in turn activates Tank-binding kinase-1 (TBK1), inducible I-κB kinase (IKK-ε) and IRF-3, which then translocates to the nucleus to facilitate transcription of IFN genes (reviewed in Gebhardt et al., 2017).

Although RIG-I and MDA5 both detect dsRNA within the cytosol, their functions are non-redundant. RIG-I recognizes relatively short dsRNA species (<1 kb) with 5′ppp or 5′pp, which are produced in certain virus infections (Hornung et al., 2006; Pichlmair et al., 2006). In contrast, MDA5 recognizes long dsRNA, which is present during picornavirus replication (Kato et al., 2008; Pichlmair et al., 2009). Thus, while RIG-I is activated during infection with flaviviruses, paramyxoviruses, influenza, and others, MDA5 is responsible for detection of picornaviruses (Gitlin et al., 2006; Kato et al., 2006; Pichlmair et al., 2009; Wang et al., 2010; Feng et al., 2012). The importance of MDA5 for control of cardioviruses was demonstrated in MDA5-deficient mice, which failed to control EMCV infection and did not efficiently produce IFN (Gitlin et al., 2006; Kato et al., 2006).

In addition to RIG-like helicases described above, which activate the MAVS pathway, another IFN-inducible RNA helicase, Moloney leukemia virus 10 homolog (MOV10) was reported to enhance IFN induction (Cuevas et al., 2016). MOV10 expression in HEK293 cells restricted EMCV replication. Interestingly, MOV10 acts through IRF-3 activation, in a RLR and MAVS-independent way and signals require IKK-ε but not TBK1. Such MAVS-independent pathways are likely not critical for global IFN production in EMCV infected mice, given the major impact of MDA5 or MAVS deficiency in mice, but they may be important in specific cell types or in conditions where the other pathways may be less potent.

## Activation Of MDA5 By Cardioviruses

Laboratory of genetics and physiology 2 (LGP2), also known as Dhx58, is also a member of the RLR family but lacks a CARD domain (Yoneyama et al., 2005). Given its structural similarity and lack of a CARD domain, LGP2 was initially thought to negatively regulate dsRNA recognition by RIG-I, as its overexpression limited IFN induction by Sendai virus and Newcastle disease virus (Rothenfusser et al., 2005). However, although the negative effect of LGP2 on RIG-I remained controversial, later studies have established that LGP2 acts as a co-activator of MDA5. Mice deficient for LGP2 were impaired in responding to RNA ligands for MDA5 or to EMCV infection (Venkataraman et al., 2007; Satoh et al., 2010). LGP2 was also shown to increase the rate of MDA5 interaction with RNA and downstream signaling by facilitating the formation of numerous, shorter MDA5 filaments (Bruns et al., 2014). Thus, it appears that LGP2 can act as both a positive and negative regulator of RLR signaling, with the outcome likely dependent on the concentration of LGP2 (Bruns and Horvath, 2015). However, recombinant MDA5 was directly activated as measured by an ATP hydrolysis assay by the replicative form of dsRNA coxsackievirus B3, showing that LGP2 is not essential for activation of MDA5 *in vitro* by dsRNA (Feng et al., 2012).

Both LGP2 and MDA5 are important for detecting cardiovirus replication. MEFs deficient for either protein produce lower amounts of IFN-β when infected with EMCV (Deddouche et al., 2014). Intriguingly, LGP2 may enhance activation of MDA5 during EMCV infection by binding to RNA complementary to the Leader (L) gene and forming a complex with MDA5. This RNA sequence from L was also shown to be a potent activator of MDA5 in the absence of virus infection (Deddouche et al., 2014).

Although dsRNA can bind and activate recombinant MDA5 in the absence of other proteins, it is likely that additional partners are required for efficient activation of MDA5 *in vivo*. For example, MDA5 is phosphorylated in resting cells, and requires dephosporylation by PP1α/γ (Wies et al., 2013; Takashima et al., 2015). Additional proteins that participate in recognition of cardiovirus dsRNA have recently been described, including a study showing that TAR RNA binding protein (TRBP) interacts with LGP2 in a yeast two-hybrid screen (Komuro et al., 2016). LGP2 was found to interact with TRBP in co-immunoprecipitation experiments and depletion of TRBP by siRNA reduced interferon production induced by TMEV and EMCV. Moreover, TRBP enhanced IFN induction by TMEV and EMCV when overexpressed. Depletion of TRBP did not affect induction of IFN by Sendai virus, which is recognized by RIG-I. This study establishes that TRBP participates in detection of cardiovirus dsRNA by LGP2/MDA5 but the mechanism and its importance *in vivo* remain to be elucidated.

As TRBP is a component of the RNAi machinery (Chendrimada et al., 2005; Haase et al., 2005), other molecules involved in RNAi were assessed for their role in dsRNA detection and protein activator of PKR (PACT) was found to also participate in activation of IFN signaling by cardioviruses. When PACT was depleted by siRNA, interferon production was decreased during infection of both TMEV and EMCV. Overexpression of PACT also increased IFN activation when LGP2 was co-expressed with MDA5. Intriguingly, single-stranded TMEV genome enhanced the association of LGP2 and PACT, which suggests that a secondary structure in the TMEV genome might facilitate this interaction (Miyamoto and Komuro, 2017). In a separate report, PACT was shown to be required for induction of IFN by EMCV but not Sendai virus. This study also demonstrated that PACT and MDA5 were recruited to dsRNA (poly(I:C)) but not single stranded RNA, and that PACT expression increased the amount of MDA5 oligomerization (Lui et al., 2017). Both TRBP and PACT have also been reported to bind to double stranded RNA-dependent protein kinase (PKR), and PACT can bind RIG-I (Park et al., 1994; Patel and Sen, 1998; Kok et al., 2011). At the present time, it is not clear if these interactions are important for mediating recognition of cardioviruses.

Yet another partner in detecting dsRNA in cardiovirus infection was recently uncovered. A cDNA screen to identify genes involved in regulating IFN signaling revealed that DHX29 expression increased transcription of an IFN-β reporter plasmid in response to high molecular weight (HMW) poly(I:C) (Zhu et al., 2018). Depletion of DHX29 resulted in decreased phosphorylation of TBK1 and IRF-3 in response to HMW poly(I:C) and EMCV as well as decreased production of IFN-β. The authors also show that DHX29 binds to MDA5 but not MAVS or RIG-I, and binding could only be detected when MDA5 was activated by EMCV or HMW poly(I:C). Mechanistically, this study demonstrated that DHX29 mediated RNA binding of MDA5 by interacting with MDA5 through its N-terminus and RNA through its DEXD and helicase domains, and that DHX29 promotes formation of MDA5 filaments, which are required for activation (Zhu et al., 2018). DHX29 was also independently described to interact with RIG-I (Sugimoto et al., 2014), although it may be of greater importance for activation of MDA5 (Zhu et al., 2018).

**FIGURE 2:**
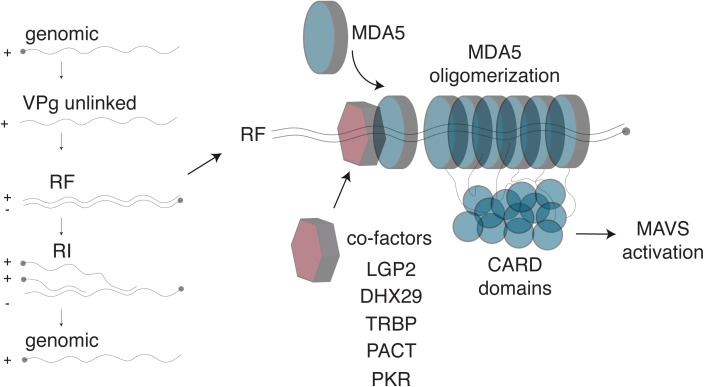
Detection of *Cardiovirus* dsRNA by MDA5. **(Left)** After genome delivery into cells, the 3B/VPg protein (closed circle) is cleaved from the 5′end of the RNA. The replicative form (RF) results from synthesis of the negative strand. The newly synthesized negative strand is then used as a template to generate new genomes, via the formation of replicative intermediates (RI). **(Right)** MDA5 was shown to detect the RF. After dsRNA recognition, MDA5 molecules oligomerize on the dsRNA molecule, exposing their N-terminal CARD domains, which oligomerize and trigger IFN transcription through MAVS activation. Several co-factors, including LGP2 and PKR, were identified that enhance Cardiovirus dsRNA recognition by MDA5, possibly by facilitating initial dsRNA recognition.

Together, these studies show that multiple proteins facilitate recognition of dsRNA by MDA5 during cardiovirus infection (**Figure 2**). Thus far, these proteins include LGP2, DHX29, PACT and TRBP. Additional helicases are also likely to participate in RLR-dsRNA complex formation but their activities remain to be clarified (Oshiumi et al., 2016). Moreover, recent discoveries have identified a novel role for PKR in recognition of dsRNA and activation of MDA5, which will be discussed below. As the number of proteins mediating MDA5 activation grows, so does the number of questions about how this pathway functions. For example, what role does each of the proteins play and how do they work together mechanistically to activate MDA5? Do they play non-redundant roles, or do they function in the same way but in different cell types? Resolving these questions will allow for deeper understanding of the first line of immune defense against RNA viruses.

## The Role Of PKR in Antiviral Responses to Cardioviruses

Upon recognition of dsRNA, PKR controls virus infection by phosphorylating eukaryotic initiation factor 2 (eIF2α), which inhibits translation (Farrell et al., 1977). In this way, an infected cell can suppress production of viral proteins. Phosphorylation of eIF2α also leads to stress granule formation and induces autophagy, and both pathways are commonly observed during virus infection (Paul and Munz, 2016; Poblete-Duran et al., 2016). Not surprisingly, many viruses have evolved strategies to inhibit the activation or function of PKR (reviewed in Garcia et al., 2007), as evidenced by the fact that PKR-deficiency did not modify the survival time of EMCV infected mice (Yang et al., 1995).

In addition to its role in inhibiting translation, recent evidence has emerged to show that PKR participates in production of IFN. For example, PKR appears to be important for nuclear translocation of IRF-3 following MDA5 activation (Pham et al., 2016). PKR was found to bind to MDA5 and this interaction was not disrupted by nuclease treatment, indicating that binding does not depend on the presence of RNA. In PKR-deficient cells, EMCV infection failed to induce IRF-3 translocation to the nucleus. Moreover, a constitutively active mutant of PKR induced IFN through MAVS. The effect of PKR on induction of IFN required its catalytic activity but did not depend on phosphorylation of eIF2α (Pham et al., 2016). In a separate report, PKR was shown to be important for normal processing of IFN-β mRNA, suggesting that PKR may function at multiple points in the IFN pathway (Schulz et al., 2010). Additionally, activation of PKR by HMW poly(I:C) was shown to be inhibited in cells depleted of MDA5 and MAVS, suggesting that MAVS influences activation of PKR. Moreover, MAVS and PKR were found to interact in co-IP experiments, and the interaction depended on the CARD domain in MAVS and the dsRNA binding domain of PKR (Zhang et al., 2014). Together, these studies clearly indicate a role for PKR in MDA5-dependent IFN induction, although several mechanisms may be involved, which require clarification.

The role of PKR in IFN production after cardiovirus infection remains to be resolved. In one study that examined Mengo virus infection, knockdown of PKR led to decreased induction of IFN-β in Hela cells, suggesting that PKR may play a role in the MDA5 pathway during cardiovirus infection (Langereis et al., 2013). This observation fits with the model proposed by Onomoto et al. (2012), which was based on Influenza virus infection and suggests that PKR triggers the formation of “antiviral stress granules” that serve as a recruitment platform for dsRNA and RIG-like helicases, thereby enhancing IFN production. PKR is, however, not essential for IFN production in cardiovirus-infected cells because Mengo virus possessing a deletion in the zinc-finger of L, which abrogates its functions as an interferon antagonist, was found to induce interferon in the cells lacking PKR and RNase L (Feng et al., 2012). Cardioviruses may also inhibit PKR through activity of the L protein, as stress granule formation was prevented by the L protein of Mengo, TMEV, and SAFV-2 during virus infection (Borghese and Michiels, 2011). However, direct inhibition of PKR by L remains to be demonstrated.

## Detection Of Extracellular dsRNA

During infection, viral dsRNA can also be released into the extracellular milieu, either non-specifically during lysis of infected cells or possibly intentionally by exocytosis to trigger innate immunity by uninfected cells. dsRNA can then be endocytosed by neighboring cells and infiltrating immune cells and lead to IFN production. TLR3 recognizes dsRNA in endosomes and signals through TRIF to activate IRF-3 and NF-κB. The importance of TLR3 in context of cardiovirus infection may depend on the model of infection. For example, mice deficient for MyD88 or TLR3 were not significantly more susceptible than wild-type mice to EMCV infection (Kato et al., 2006). In a separate report, however, TLR3-deficient mice had higher viral loads in the liver and heart and were more susceptible to infection (Hardarson et al., 2007). Also, a study that evaluated the role of TLR3 in controlling a strain of EMCV with tropism for β cells of the pancreas found that TLR3 protected mice from a fatal infection and that TLR3-deficent mice produced less IFN-β early in infection (15 and 18 h post-infection). However, this deficiency was transient and mice lacking TLR3 produced levels of IFN-β equivalent to wild-type animals at 24 h post-infection. In the same experiments, the authors demonstrated that mice lacking MDA5 succumbed to the infection more rapidly than TLR3-deficient mice and produced less IFN-β at 24 h post-infection (McCartney et al., 2011). Finally, a recent report using intracerebral inoculation of the GDVII strain of TMEV evaluated the importance of these molecules for control of virus replication and induction of IFN. Trif-/-, MyD88-/-, and mice lacking both Trif and MyD88 showed wild-type levels of IFN induction, while MAVS-deficient animals were slightly but significantly impaired. When Trif, MyD88 and MAVS were all depleted, however, mice were unable to induce IFN. Only mice lacking MyD88 and Trif, or mice lacking MyD88, Trif and MAVS showed increased titers of GDVII in the brain (Pfefferkorn et al., 2016). Thus, both TLR3 and RLRs contribute to controlling virus replication *in vivo*, although the relative importance of these pathways may depend on the virus and route of inoculation.

Surprisingly, MDA5 is also responsible for the vast majority of IFN produced from extracellular dsRNA *in vivo.* Mice deficient for MDA5 produced substantially less IFN when administered polyI:C, whereas TLR3-deficient mice responded like wild-type (Gitlin et al., 2006). These results raise the question of how dsRNA taken up through endocytosis could gain access to the cytoplasm. The mechanism by which dsRNA could be internalized and then access the cytoplasmic RLRs has been unresolved until a recent discovery identified SIDT2, the mammalian ortholog of the SID-1 dsRNA transporter in *Caenorhabditis elegans*, as a transporter of dsRNA from the endosome to the cytoplasm. SIDT2 was shown to be important for mediating detection of dsRNA in the context of EMCV infection *in vivo*. Mice that were deficient for SIDT2 failed to control replication, produced less IFN-β, and succumbed to infection (Nguyen et al., 2017). These data suggest that a crucial pathway for innate signaling in EMCV infection is release of viral RNA into the extracellular milieu, endocytosis, and subsequent transfer of viral RNA to the cytoplasm to access MDA5.

## Inhibition Of Interferon Production By Cardioviruses

Two proteins encoded by cardioviruses were shown to counteract IFN production in infected cells: protease 3C, which is responsible for the processing of the virus-encoded polyprotein, and the leader protein (L), which corresponds to the N-terminal peptide of the polyprotein.

**FIGURE 3:**
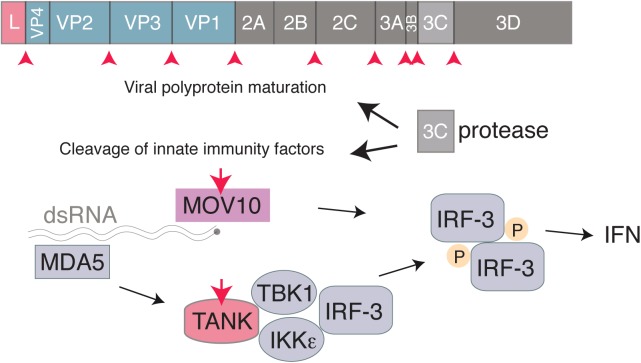
Interferon antagonism by the *Cardiovirus* 3C protease. The 3C protease is responsible for cleavage of the Cardiovirus polyprotein produced from translation of the genome. In addition, the Cardiovirus 3C protease cleaves host proteins, such as TANK and MOV10, to prevent the cell from producing IFN.

3C is a cysteine proteinase with a trypsin-like serine protease fold, responsible for most cleavages occuring during the maturation of the viral polyprotein (Pelham, 1978). Like 3C proteases of other picornaviruses that were shown to target critical factors involved in IFN induction such as RIG-I (Barral et al., 2009), EMCV 3C was reported to cleave TRAF family member-associated NF-kB activator (TANK) in infected cells, thus disrupting the complex involving TBK1, IKKe and IRF3 and limiting type I IFN production (Huang et al., 2017). Likewise, EMCV 3C was shown to target MOV10, an RNA helicase that acts in a MAVS-independent way, as a possible innate immune evasion mechanism (Cuevas et al., 2016; **Figure 3**).

L is a small, multifunctional protein of 67–76 amino acids expressed by all cardioviruses (**Figure 1**). L contains an N-terminal zinc finger motif (Cys-His-Cys-Cys), an acidic domain, a serine/threonine rich domain, and a C-terminal Theilo domain, which is present in SAFV and TMEV but absent in EMCV. L was shown to be dispensable for replication of TMEV in cell culture but its loss inhibits spread in cells that have a functional interferon response, like L929 cells, and also impairs the viral persistence *in vivo* (van Pesch et al., 2001). L deletions in EMCV prevent the virus from shutting off host protein synthesis and enable interferon production (Zoll et al., 1996, 2002). Mengo virus containing a mutation in the zinc finger of L failed to inhibit IFN synthesis and its replication was inhibited during low MOI infections *in vitro*. In mice lacking the IFN α/β receptor, the mutant virus behaved as wild-type, but in wild-type mice, replication of the L mutant virus was impaired and it failed to cause disease, demonstrating that activity of L is important for pathogenesis *in vivo* (Hato et al., 2007). Mutations in the zinc finger domain or the Theilo domain of TMEV or SAFV L inhibit its ability to antagonize interferon signaling (Ricour et al., 2009).

**FIGURE 4:**
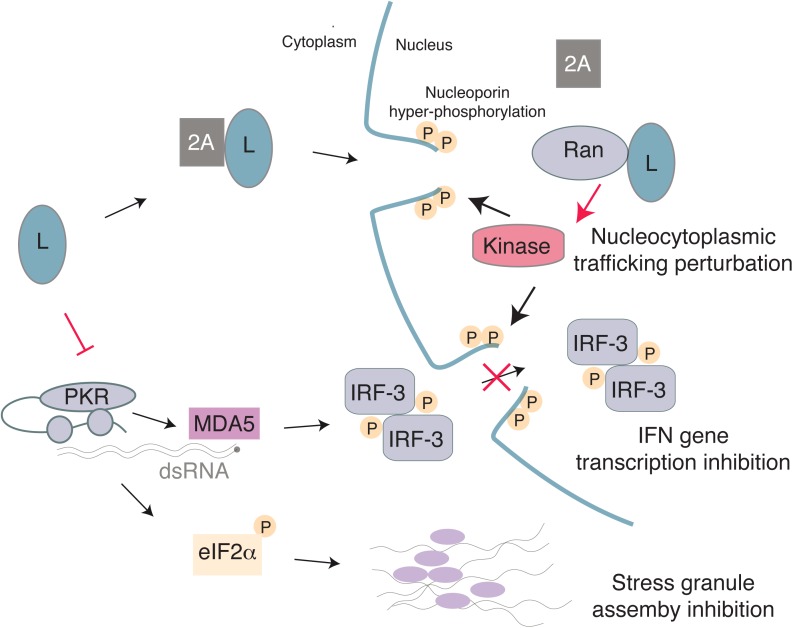
Model for the multiple activities of the *Cardiovirus* L protein. L binds 2A with low-affinity and the complex may enter the nucleus due to a NLS in 2A. Once in the nucleus, L interacts with high affinity with Ran GTPase, thus displacing 2A. The L-Ran complex would activate a kinase and trigger nucleoporin hyper-phosphorylation, thereby leading to nuclear pore complex dismantling and to nucleocytoplasmic trafficking perturbation. On the other hand, L may inhibit PKR, thus preventing translation arrest through eIF2α phosphorylation and therefore block assembly of stress granules. Inhibition of IFN gene transcription by L may result from IRF-3 trafficking perturbation and/or from the absence of PKR-enhanced dsRNA detection by MDA5.

A critical step in production of IFN following detection of viral replication by MDA5 and other molecules is nuclear translocation of IRF-3 and NF-κB. These proteins enable transcription of the IFN genes to produce mRNA, which must then be exported from the nucleus for translation. Since picornaviruses do not replicate within the nucleus, many viruses within this family disrupt nucleocytoplasmic trafficking, which results in inhibition of IFN production and translocation of nuclear proteins to the cytosol to benefit viral replication (reviewed in Yarbrough et al., 2014; Flather and Semler, 2015). The mechanism of how L interferes with IFN production may be due to its abilities to disrupt nucleocytoplasmic trafficking, activation of IRF-3, and assembly of stress granules in infected cells (**Figure 4**). Each of these activities will be explored below.

The L protein of cardioviruses perturbs the function of the nuclear pore complex (NPC) (Porter et al., 2006). In mammals, the NPC consists of approximately 30 different proteins, called nucleoporins (Nups) and enables transit across the nuclear membrane (Gorlich and Kutay, 1999; Wente and Rout, 2010). While small molecules and ions are able to diffuse through the NPC, molecules larger than approximately 20–40 kDa require active transport, which is regulated by transport receptors called karyopherins (Yarbrough et al., 2014). Transport into the nucleus requires a short amino acid motif, called a nuclear localization sequence (NLS) that can interact with either the α or β subtypes of karyopherins, depending on the sequence of the protein’s NLS. Binding and dissociation of NLS-containing proteins by karyopherins is also regulated by small GTPase Ran. In the cytosol, Ran is bound to GDP and can bind cargo proteins. Once in the nucleus, however, Ran is converted to the GTP bound form by the Ran guanine nucleotide exchange factor (RanGEF) and dissociates from cargo. Export then requires a nuclear export sequence (NES) that binds to karyopherins bound to RanGTP, and dissociation of this complex occurs in the cytoplasm when a Ran GTPase-activating protein (RanGAP) hydrolyzes GTP to GDP. In this way, the RanGDP/GTP gradient regulates directional transport into and out of the nucleus.

Localization of L to the nucleus depends on expression of 2A, which contains a NLS in its C-terminus (Groppo et al., 2011). Upon nuclear localization, L interacts with Ran with high affinity and 2A is displaced as the binding sites for 2A and Ran partially overlap (Petty et al., 2014). L from EMCV, TMEV, and SAFV induce hyper-phosphorylation of Nups including Nup62 and Nup98 (Ricour et al., 2009; Ciomperlik et al., 2015), likely by recruiting and activating a kinase, which may be facilitated by L binding of exportins Crm1 and CAS (Ciomperlik et al., 2016). Chemical inhibition of ERK and p38 was able to block L-mediated hyper-phosphorylation of Nups (Porter et al., 2010). Additionally, L of EMCV is phosphorylated by casein kinase 2 (CK2) and this phosphorylation is required for Nup phosphorylation, although CK2 did not phosphorylate L of SAFV or TMEV (Basta et al., 2014). It is possible, although it remains to be shown, that these kinases also play a role in inhibition of nucleocytoplasmic trafficking by L of TMEV and SAFV.

In addition to its role in disrupting nucleocytoplasmic trafficking, TMEV and Mengo L prevent production of type I IFN in infected cells by interfering with IRF-3 dimerization and TMEV L also prevents export of mRNA from the nucleus (Delhaye et al., 2004; Ricour et al., 2009). For both TMEV and Mengo virus, dimerization of IRF-3 was impaired despite the protein having been phosphorylated. Inactivation of IRF-3 occurs despite reports that it accumulates in the nucleus of infected cells (Delhaye et al., 2004). These data suggest that dsRNA is detected in cardiovirus infected cells leading to activation of MAVS and downstream kinases, but that IRF-3 is unable to induce IFN transcription.

Stress granules can form in cells during virus infection and often result from inhibition of translation following phosphorylation of eIF2α by PKR (White and Lloyd, 2012). The L protein of Mengo, TMEV, and SAFV-2 inhibits stress granule formation during infection and ectopic expression of L was able to prevent thapsigargin- and arsenite-induced stress granules (Borghese and Michiels, 2011). Stress granules formed during infection with viruses containing deletions in the zinc-finger domain or a mutation in the Theilo domain of L, indicating that these motifs are also important for inhibition of stress granules (Borghese and Michiels, 2011).

While L inhibits nucleocytoplasmic trafficking, stress granule formation, and possibly PKR activation, it has not been possible to uncouple these events using L mutants. When one function of L is disrupted, all functions are simultaneously impaired. Therefore, it remains possible that L inhibits IFN production by blocking PKR activation, by interfering with IRF-3 dimerization or nucleocytoplasmic trafficking, or through a combination of these mechanisms (**Figure 4**).

The importance of these antiviral pathways in controlling infection is underscored by the variety of mechanisms that viruses have evolved to prevent their activity. For example, the L protein of foot-and-mouth disease virus (FMDV), a picornavirus in the genus *Aphthovirus*, has proteolytic activity whereas the L protein of cardioviruses does not. Despite the major differences in these proteins, they all still function to inhibit induction of IFN. FMDV L^pro^ can cleave eIF4G (Devaney et al., 1988; Kirchweger et al., 1994; Guarne et al., 1998) and therefore reduce translation of cellular mRNAs, and can also perturb IFN transcription by cleaving NF-κB (de Los Santos et al., 2006, 2007, 2009). However, in the context of a chimeric Mengo virus infection, FMDV L^pro^ was less effective at inhibiting IFN induction *in vitro* and *in vivo* (Hato et al., 2010). Similar convergent evolution is apparent when considering the 2A protein of picornaviruses. Whereas 2A functions as a protease for most picornaviruses and cleaves mediators of type I interferon signaling, this activity is not present in cardioviruses. Nevertheless, L still targets some of these same molecules for inactivation (Agol and Gmyl, 2010). Additionally, both 2A of enteroviruses and L of cardioviruses inhibit stress granule assembly (Yang et al., 2018).

The functions of L appear to be sufficiently important to the virus so that it maintains high levels of L expression throughout infection. EMCV and TMEV undergo a frameshift during translation later in infection by 2A binding to a stem-loop structure in the genome (Napthine et al., 2017). This frameshift decreases expression of non-structural proteins 2BC-3ABCD by 74–82% (Finch et al., 2015). A follow up study using metabolic labeling estimated the frameshifting to be 46–76% efficient (Ling and Firth, 2017). This mechanism may allow for cardioviruses, and perhaps other picornaviruses, to increase the translation of structural proteins later in infection. Due to its position in the genome, however, L expression would remain high throughout infection despite it not having a structural role for virus assembly. Thus, it may be important for cardioviruses to express sufficient levels of L to counteract the immune response throughout the replication cycle.

## What Cells are Most Important for IFN Production During Cardiovirus Infection?

With effective ways to inhibit the production of interferon during infection, control of cardioviruses likely depends on nearby uninfected cells to produce interferon. Intriguingly, these pathways seem to also depend on MDA5, although TLR3 may also be important in certain cell types such as plasmacytoid dendritic cells (Hornung et al., 2006). As discussed, recent data suggest a model where viral dsRNA is released, endocytosed, and then the RNA is translocated to the cytosol where it is detected by MDA5.

In the CNS, astrocytes appear to be the primary producers of IFN-β for several neurotropic viruses that preferentially infect neurons, such as TMEV and La Crosse virus (Kallfass et al., 2012; Pfefferkorn et al., 2016). Using transgenic mice that expressed firefly luciferase under the control of the IFN-β promoter restricted to different cell types, the authors were able to determine that 73% of IFN-β production during a neurotropic TMEV infection was from astrocytes, whereas only 1% was from neurons, which are the primary target of infection. In mice lacking MAVS, IFN-β production was slightly but significantly reduced, suggesting that the RLR pathway is active during infection but may not be the only pathway activated by TMEV in astrocytes. Intriguingly, mice deficient for MyD88 and Trif did not show a significant decrease in IFN-β induction, although induction of IFN-β was completely abrogated in mice deficient for MAVS, MyD88 and Trif. Therefore, it appears that both RLR and TLR signaling are important for IFN-β production after TMEV infection of the CNS.

Astrocytes were also shown to be primary producers of IFN during infection with rabies virus and vesicular stomatitis virus. In the case of rabies virus, astrocytes are stimulated to produce IFN by an abortive infection. That the virus is unable to replicate fully may prevent expression of viral interferon antagonists and allow for robust production of IFN. How viral replication is prevented in these cells will be important to uncover and may lead to novel insights about viral control *in vivo*. It is likely that abortive infection of astrocytes occurs during infection by TMEV. However, this remains to be demonstrated and viral RNA may well be encountered by other means.

MDA5 is critical for induction of IFN against cardioviruses in the periphery as well. *Ex vivo*, cells such as macrophages, conventional dendritic cells and fibroblasts depend on MAVS for production of IFN in response to dsRNA (Sun et al., 2006). Similarly, MDA5 was shown to be essential in these cells for type I IFN production after EMCV infection, in contrast to pDCs which induce IFN production in a TLR-dependent fashion (Gitlin et al., 2006; Kato et al., 2006). After EMCV infection of mice, some IFN is produced through TLRs, likely by pDCs, but most IFN was produced by MDA5 activation (Gitlin et al., 2006; Kato et al., 2006). Levels of IFN-I were strongly decreased in the serum of MDA5-deficient mice infected by EMCV. Whereas MDA5 expression strongly influenced survival in response to infection, the effect of MyD88 depletion had a modest effect and loss of Trif or RIG-I did not affect survival (Kato et al., 2006). Thus, MDA5 is essential for controlling EMCV infection in the periphery.

## Cardiovirus Inhibition Of IFN Effectors

Interferon secreted by infected cells binds to its receptor on surrounding cells, activating a signaling cascade that leads to expression of hundreds of interferon-stimulated genes (ISGs). Two of these ISGs, PKR and oligoadenylate synthetases (OAS) are part of the best-characterized interferon effector pathways.

As described earlier, PKR is likely antagonized by the L protein, as L inhibits PKR-induced stress granule assembly. Moreover, a recent study reported increased SUMO3 conjugation of PKR in EMCV-infected cells, which dampens PKR activation and promotes caspase-dependent PKR degradation (Maarifi et al., 2018).

**FIGURE 5:**
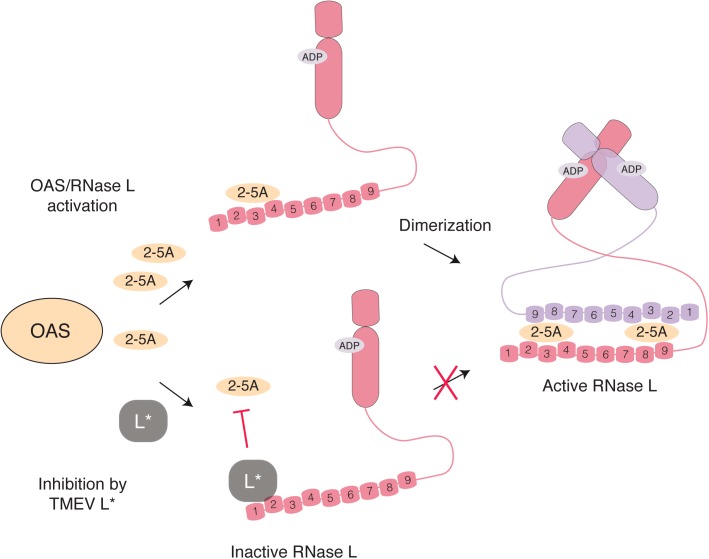
Inhibition of RNase L activation by L^∗^ TMEV L^∗^ binds RNase L ankyrin repeats 1 and 2 (numbered) through a direct protein-protein interaction, thereby preventing association of 2-5A with RNase L monomers and the consequent dimerization and activation of the enzyme.

Oligoadenylate synthetases are enzymes responsible for RNase L activation. Cardioviruses have evolved two strategies to interfere with the OAS-RNase L pathway. In an infected cell, OAS are activated by dsRNA and produce 2′-5′ oligoadenylates (2-5A). Binding of two 2-5A molecules to the ankyrin domain of the latent endoribonuclease RNase L triggers its dimerization and activation (**Figure 5**). Active RNase L then cleaves viral and cellular ssRNA leading to decreased viral replication and ultimately to apoptosis of the cell. Interestingly, RNA fragments generated by RNase L can amplify IFN production in a RIG-I, MDA5 and MAVS-dependent way (Malathi et al., 2007). RNase L targets both viral and cellular mRNA but is also predicted to cleave the genome of ssRNA viruses, as reported for EMCV (Li et al., 1998). In addition to 2′-phosphodiesterases and phosphatases that tightly regulate the system by degrading 2-5A within minutes of their synthesis, RNase L activity can be negatively regulated by the RNase L inhibitor (RLI/ABCE) (Bisbal et al., 1995).

RLI/ABCE expression is induced by EMCV and correlates with RNase L inhibition (Martinand et al., 1999). Accordingly, overexpression of RLI/ABCE inhibited the action of IFN against EMCV (Bisbal et al., 1995). RNase L inhibition by EMCV-induced RLI is, however, partial as RNase L antiviral activity against EMCV was demonstrated *in vitro* using dominant negative RNase L and OAS1 overexpression (Chebath et al., 1987; Zhou et al., 1998) and *in vivo*, in RNase L-deficient mice, which presented increased EMCV infection and mortality compared to wild-type mice (Zhou et al., 1997).

The L^∗^ protein of TMEV was found to potently inhibit RNase L through a direct protein-protein interaction (Sorgeloos et al., 2013). Mechanistically, L^∗^ binds to RNase L ankyrin repeats 1 and 2, thereby preventing 2-5A binding to the enzyme and further activation steps (Drappier et al., 2018; **Figure 5**). In wild-type macrophages, replication of L^∗^-mutant was significantly impaired as compared to that of the wild-type virus (Sorgeloos et al., 2013). In contrast, L^∗^-mutant and wild-type viruses replicated to the same level in RNase L-deficient primary peritoneal macrophages. Moreover, L^∗^ was shown to be active *in vivo* in the context of MHV chimeric viruses; L^∗^ could substitute for another viral RNase L inhibitor, namely the ns2 phosphodiesterase of MHV, in the liver of infected mice (Drappier et al., 2018). The fact that the virus devotes one of its proteins to RNase L antagonism highlights the importance of this antiviral pathway against TMEV. Interestingly RNase L inhibition by L^∗^ is highly species-specific; L^∗^ of a mouse TMEV strain inhibits mouse RNase L but not its orthologs from other species including rat (Sorgeloos et al., 2013; Drappier et al., 2018). Accordingly, L^∗^ of a rat TMEV strain inhibits rat but not mouse RNase L.

Theiler’s murine encephalomyelitis virus is the only cardiovirus expressing L^∗^, and thus the only cardiovirus known to directly inhibit RNase L. This could stem from its tropism for macrophages, which are the main TMEV target during the chronic phase of infection and in which the OAS-RNase L system is particularly active (Zhao et al., 2012). However, macrophages were reported to play important roles in EMCV pathogenesis, including for viral replication and dissemination in piglets (Papaioannou et al., 2003) and as reservoir cells for EMCV persistence in rats (Psalla et al., 2006). Since EMCV is sensitive to RNase L activity, it is possible that another EMCV protein will have developed some RNase L antagonistic activity, which might be identified using the appropriate host-pathogen context. Interactions between SAFVs and RNase L have yet to be described, but it is also likely that these viruses have evolved ways of inhibiting this pathway.

## Conclusion and Future Perspectives

Given the many ways that picornaviruses inhibit interferon production and signaling in infected cells, it is not surprising that the most important producers of IFN would be uninfected or abortively infected cells. Indeed, cardioviruses efficiently block IFN in infected cells but loss of MDA5 in mice causes them to be more susceptible to virus infection. These data indicate that detection of cytoplasmic dsRNA by MDA5 occurs in cells that are not productively infected (Gitlin et al., 2006) and the recent finding that SIDT2 mediates this process opens many new exciting areas of research (Nguyen et al., 2017). Which molecules might be important for release of viral RNA? Is there a role for exosomes in this process? How might these pathways be stimulated pharmacologically? Preventing release of viral RNA and subsequent detection by uninfected cells may represent selective pressure favoring non-lytic release. Given the exquisite genetic malleability in response to natural selection displayed by viruses, it is likely that viruses will have evolved mechanisms of inhibiting detection of viral RNA by uninfected cells, perhaps by restricting dsRNA release or by secreting proteins that inhibit RNA transport into uninfected cells. It will be exciting to see how discoveries unfold in this area of research.

Several recent studies involving cardioviruses have revealed a more complicated picture regarding initial detection of replicating RNA and induction of IFN. While it is clear that the helicases LGP2, DHX29, PACT and TRBP work in concert with MDA5 for detection of dsRNA, it remains to be determined how these molecules coordinate and interact and whether they function in a cell-type specific manner. It will also be important to resolve the way in which PKR functions to activate IFN signaling. Future studies in this area will likely have broad relevance for innate detection of viruses.

Finally, the mechanisms by which L disrupts nucleocytoplasmic trafficking, stress granule formation, and interferon production clearly require further clarification. Mutational analysis of L has revealed that these activities are tightly coupled, suggesting that the L interacts with protein(s) that can serve as a common node in each of these pathways. As IFN signaling and stress granules are important for a variety of viral pathogens, answers to these questions may provide broadly relevant insight into host-pathogen interactions.

## Author Contributions

EF, MD, and TM wrote the manuscript and approved its final version.

## Conflict of Interest Statement

The authors declare that the research was conducted in the absence of any commercial or financial relationships that could be construed as a potential conflict of interest.
